# QuickStats

**Published:** 2014-10-03

**Authors:** FROM THE NATIONAL CENTER FOR HEALTH STATISTICS

**Figure:**
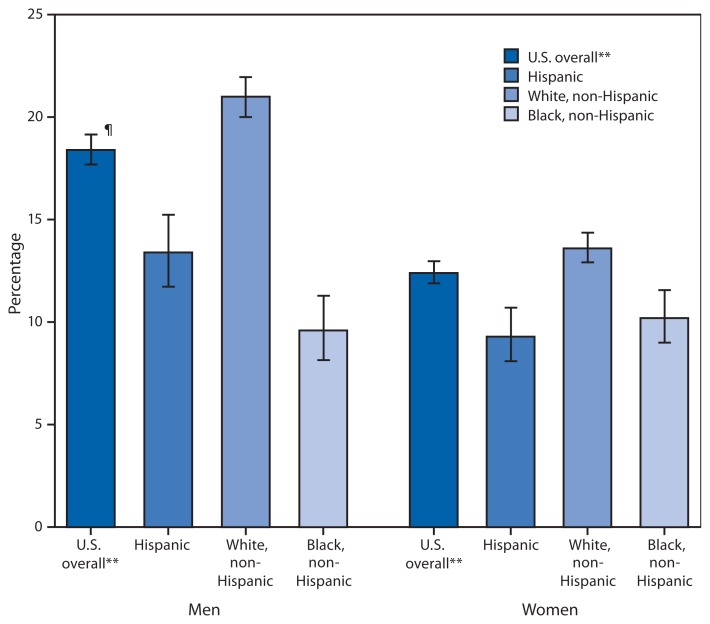
Percentage of Adults Aged ≥18 Years with Trouble Hearing,* by Sex and Race/Ethnicity^†^ — National Health Interview Survey, United States, 2012^§^ * Based on responses to the following question: “Without the use of hearing aids or other listening devices, is your hearing excellent, good, a little trouble hearing, moderate trouble, a lot of trouble, or are you deaf?” For this figure, “a little trouble hearing,” “moderate trouble,” “a lot of trouble, “ and “deaf” are combined into a single category, “trouble hearing.” Unknowns were not included in the denominators when calculating percentages of “trouble hearing.” ^†^ Refers to persons who are of Hispanic ethnicity and might be of any race or combination of races. “Non-Hispanic” refers to all persons who are not of Hispanic ethnicity, regardless of race. ^§^ Estimates are based on household interviews of a sample of the noninstitutionalized U.S. civilian population and are age adjusted to the projected 2000 U.S. population as the standard population using four age groups: 18-44, 45-64, 65-74, and ≥75 years. ^¶^ 95% confidence interval. ** Includes other races/ethnicities not shown separately.

Overall, in 2012, non-Hispanic white adults were more likely to report having trouble hearing compared with Hispanic adults and non-Hispanic black adults. Men (18%) were more likely to report having trouble hearing than women (12%). Among Hispanic and non-Hispanic white adults, men were more likely to report having trouble hearing; however, this pattern was not observed for non-Hispanic black adults, among whom no statistically significant difference was observed between men and women.

**Source:** Blackwell DL, Lucas JW, Clarke TC. Summary health statistics for U.S. adults: National Health Interview Survey, 2012. Vital Health Stat 2014;10(260). Available at http://www.cdc.gov/nchs/data/series/sr_10/sr10_260.pdf.

**Reported by:** Jacqueline W. Lucas, MPH, jacqueline.lucas@cdc.hhs.gov, 301-458-4355; Tainya C. Clarke, PhD; Debra Blackwell, PhD.

